# Immune Checkpoint and Anti-Angiogenic Antibodies for the Treatment of Non-Small Cell Lung Cancer in the European Union and United States

**DOI:** 10.3390/pharmaceutics13060912

**Published:** 2021-06-21

**Authors:** Marion Ferreira, Thomas Secher, Nathalie Heuze-Vourc’H, Karen L Reckamp

**Affiliations:** 1 INSERM, Centre d’Etude des Pathologies Respiratoires, U1100, Boulevard Tonnellé, F-37032 Tours, France; secher.thomas@gmail.com (T.S.); nathalie.vourch@univ-tours.fr (N.H.-V.); 2 Faculté de Médecine, Université de Tours, F-37032 Tours, France; 3 CHRU de Tours, Département de Pneumologie et Explorations fonctionnelles Respiratoires, F-37032 Tours, France; 4 Department of Medicine, Cedars Sinai Medical Center, Los Angeles, CA 90048, USA; Karen.Reckamp@cshs.org

**Keywords:** lung cancer, immune checkpoint inhibitors, anti-angiogenic, antibodies, cancer treatment

## Abstract

Several types of antibodies (Abs) are currently used in non-small cell lung cancer (NSCLC). Anti-angiogenic and immune checkpoint inhibitor (ICI) Abs are the most frequent treatments used alone or with chemotherapy in metastatic NSCLC, for the front line and beyond. Considering the many therapeutic options for locally advanced and metastatic lung cancer and differences in use according to geographic area, we present here a comprehensive review of the marketed ICI and anti-angiogenic Abs approved in the European Union (EU) and the US to treat locally advanced and metastatic NSCLC patients. We briefly describe the different molecules and their development in thoracic oncology and compare pharmacokinetic data, processing decision algorithms and marketing authorizations by the EMA and US Food and Drug Administration (FDA).

## 1. Introduction

Lung cancer is responsible for significant morbidity and mortality worldwide; it is the leading cause of cancer death. It comprises non-small cell lung cancer (NSCLC), the most frequent subtype, and small cell lung cancer (SCLC). Despite recent therapeutic progress, it still constitutes a major public health problem, due to diagnoses frequently being made at an advanced stage with a high cost to society. A better understanding of tumor and immune signaling pathways has enabled the recent discovery of new molecular targets. Immunotherapy with immune checkpoint inhibitors (ICIs) has changed the treatment paradigm in many tumor types and improved survival in a subset of patients with advanced or metastatic cancers [1]. The choice of standard therapeutic options for patients with locally advanced and metastatic NSCLC has been expanded by monoclonal antibodies (Abs) like ICIs, but also anti-angiogenic Abs and other targeted Abs [2]. 

Several types of Abs are currently used for the treatment of NSCLC, but this review will discuss ICI and anti-angiogenic Abs. In fact, some Abs are directed to the epidermal growth factor receptor (*EGFR*), such as necitumumab, a monoclonal antibody (Ab) which blocks the interaction between *EGFR* and its ligands. It was approved by the Food and Drug Administration (FDA) in 2015 for first-line treatment of metastatic squamous NSCLC in combination with chemotherapy [3] but the National Comprehensive Cancer Network (NCCN) guidelines recommend against the use of this Ab, based on its toxicity, cost, and limited improvement in efficacy when compared to chemotherapy. 

We will discuss commonly used and marketed ICIs for patients with NSCLC, including nivolumab [4,5,6,7], pembrolizumab [8,9,10,11], cemiplimab [12], durvalumab [13], atezolizumab [14,15,16], and ipilimumab [6,17]. They act by targeting immune checkpoints expressed by tumor infiltrating lymphocytes (TILs)—programmed-death 1 (PD-1) or cytotoxic T-lymphocyte-associated protein 4 (CTLA-4)—or expressed by cancer and tumor infiltrating immune cells—programmed-death ligand 1 (PD-L1) [18]. Anti-angiogenic Abs used in NSCLC are bevacizumab [19,20,21] and ramucirumab [22,23]. The choice of ICI molecule depends on the expression of tumor PD-L1 for some molecules or associations and the choice of anti-angiogenic therapy is based on patients’ contraindications and tumor histology. ICIs have proven to be better tolerated than chemotherapy [5,9], but the response to single-agent ICIs is not durable in most patients and only a minority have a durable benefit [14,24]. Even if ICIs represent one of the most promising therapeutic approaches in metastatic NSCLC, combination treatments are frequently used including chemotherapy, multiple ICIs or ICIs and other Abs, in order to improve their efficacy.

Considering the various therapeutic options for locally advanced and metastatic lung cancer and differences in use according to geographic area, we present a comprehensive review of the marketed ICI and anti-angiogenic Abs approved in the European Union (EU) and the US to treat NSCLC patients. We will focus our discussion on NSCLC without oncogenic drivers except for anti-angiogenic Abs, which can be used in combination with tyrosine kinase inhibitors (TKI) in case of *EGFR*-mutated tumors. The European Medicines Agency (EMA) is a decentralized agency of the EU responsible for the scientific evaluation, supervision, and safety monitoring of medicines. It serves the EU and other countries: Iceland, Norway, and Liechtenstein. United Kingdom (UK) and Switzerland are therefore not affected by this review. Even before its withdrawal from the EU, the UK did not use EMA recommendations for its drug approvals; Switzerland has its own marketing authorization system (Swissmedic’s). It is noteworthy that a marketing authorization may be obtained through different procedures: (1) the centralized procedure which allows the marketing of a drug on the basis of a single assessment at the EU level; (2) the decentralized procedure whereby a medicine can be authorized simultaneously in several EU member countries; (3) the mutual recognition procedure through which companies whose medicinal product is authorized in one member country can apply for the recognition of this authorization in other EU countries. Finally, it is possible to obtain a (4) national authorization: while the majority of new and innovative medicines are evaluated by the EMA and authorized by the European Commission in order to be marketed in the EU, most generic medicines and medicines available without a prescription are assessed and authorized nationally in the EU. This therefore implies that there is no complete uniformity within the EU and it is possible that there are certain particularities depending on the country (details available on ema.europa.eu: *The European system of drug regulation* (accessed on)*)*. Here, we examine the authorizations of the EMA, which are in the majority of cases, for anticancer antibodies in NSCLC, those found in the member states; we stopped the analysis of authorizations in May 2021. Importantly, the US FDA Oncology Center of excellence launched in 2019 a project called ”Or-bis”, which aims to accelerate the procedures for granting marketing authorizations for drugs throughout the world. It includes the health authorities of Singapore, Australia, Brazil, Switzerland, Canada, and the United Kingdom (details available on https://www.fda.gov/about-fda/oncology-center-excellence/project-orbis, accessed on 1 May 2021).

Herein, we briefly describe the different ICI and anti-angiogenic Ab agents and their development in thoracic oncology, and compare pharmacokinetic data and marketing authorizations by the EMA and FDA.

## 2. Immune Checkpoint Inhibitors 

In addition to the characteristics of the tumor cell, the growth and metastatic potential of cancer are also dependent on interactions with the immune system [25]. ‘Checkpoints’ allow the immune system to avoid unwanted damage to adjacent tissues possibly caused by activated T cells; these ‘checkpoints’ are used to modulate the duration and amplitude of immune responses [26]. These mechanisms make it possible to limit the immune response and prevent damage caused by excessive autoimmunity or inflammation. These are the co-inhibitor receptors (TIGIT, TIM-3, etc.) which, via upregulation, will act to deactivate the activated T cells. Thus, the balance between co-stimulating and co-inhibiting signals will determine the fate of activated T cells [18]. In cancer, T-cells primed to respond to tumor cells are exposed continuously to tumor antigens during active malignancy, which may result in upregulation of multiple inhibitory receptors, culminating in decreased activity against tumor cells, a phenomenon known as T-cell exhaustion [27]. These multifaceted interactions can be manipulated to drive effective anticancer immunity. For example, several agonist antibodies targeting immune co-stimulatory receptors (CD27, CD40, OX40) have been evaluated clinically in cancer for several years, but none have been approved to date, nor have they begun any phase III randomized trials [28]. On the other hand, T cell exhaustion could be overcome by modulating the inhibitory pathways with antagonist Abs [27]. 

### 2.1. Anti-CTLA-4 Antibodies

**Figure 1:**
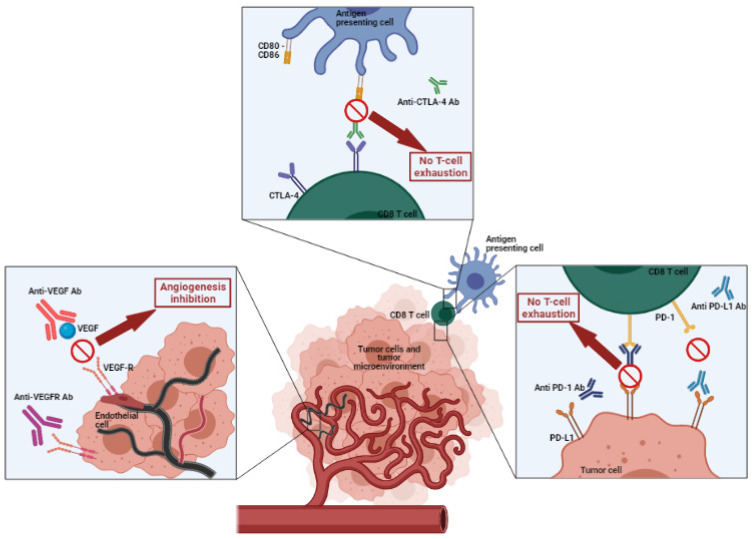
Characteristics of immune checkpoint inhibitors and anti-angiogenic antibodies used in non-small cell lung cancer (NSCLC). Created with BioRender.com.

CTLA-4 is a protein receptor located on the cell membrane of T lymphocytes. It acts as a switch—inhibiting the action of the lymphocyte—when it comes into contact with the CD80 or CD86 proteins on the surface of a cell presenting antigen. Abs to CTLA-4 block the inhibition of CD80/CD86-dependent T cell activation and in turn prolong anti-tumor activity (Figure 1). 

In lung cancer, only one anti-CTLA-4 antibody is currently used: ipilimumab; a fully human IgG1 kappa monoclonal (Table 1). The efficiency of ICIs targeting CTLA-4 were initially demonstrated for the treatment of metastatic melanoma [17,29] before being expanded in metastatic lung cancer.

### 2.2. Anti-PD-1 and PD-L1 Antibodies 

One mechanism of immune suppression in NSCLC is the expression of inhibitory molecules in the tumor microenvironment. PD-L1 (B7-H1) is expressed on tumor cells in approximately half of NSCLC; its expression can contribute to poor prognosis by suppressing T cell function and promoting tumor cell immune escape [31]. Both PD-L1 and PD-L2 (B7-DC) bind to PD-1 but PD-L1 expression is predominantly confined to the tumor microenvironment in contrast to PD-L2, which is mainly expressed on dendritic cells and macrophages [32] (Figure 1). To better understand how ICIs work, one should understand that when the link is made between the PD-1 receptor present on cytotoxic T lymphocytes and the PD-L1 ligand located on tumor cells, it creates a shield, making the tumor cell invisible to T lymphocytes. This is responsible for the phenomenon of T lymphocyte exhaustion. By using anti-PD-1 or anti-PD-L1 antibodies, binding is prevented and the cytotoxic anti-tumor function of T lymphocytes becomes active, generating tumor cell lysis [18].

PD-1 checkpoint blockade uses Abs directed against either the receptor (PD-1) or its ligand (PD-L1). Early in the development of these therapies, two anti-PD-1 Abs were investigated and are currently used in metastatic lung cancer. Nivolumab is a fully human IgG4 monoclonal antibody targeting PD-1 (Table 1) and pembrolizumab is a humanized IgG4 antibody targeting PD-1 (Table 1). Recently, another antibody has been approved in the US: cemiplimab is a highly potent, fully human IgG4, directed against PD-1 [12].

There are currently two anti-PD-L1 antibodies that are used in locally advanced or metastatic lung cancer: atezolizumab, a humanized IgG1 antibody targeting PD-L1 (Table 1) and durvalumab, a fully human IgG1 kappa antibody targeting also PD-L1 (Table 1).

## 3. Anti-Angiogenic Antibodies

Angiogenesis is frequently upregulated in malignant solid tumors and is critical for tumor growth, proliferation, and metastases [33]. The vascular endothelial growth factors (VEGF-A, -B, -C, and -D) and their transmembrane tyrosine kinase receptors (VEGFR-1, -2, and -3) are critical pro-angiogenic factors in malignant tissues [34] (Figure 1). In NSCLC, increased VEGF expression is common and associated with adverse clinical outcomes. 

Two Abs are used in lung cancer: bevacizumab, a recombinant humanized monoclonal IgG1 antibody that neutralizes VEGF’s biologic activity through a steric inhibition of its binding to VEGF receptor; and ramucirumab, a recombinant human monoclonal IgG1 antibody with high affinity for the extracellular domain of VEGFR-2, inhibiting ligand binding (VEGF-A, -C, and -D) and activation of downstream pro-angiogenic pathways [33] (Table 1).

## 4. Pharmacokinetics (PK) and Precaution for Use of Antibodies

### 4.1. PK and Precaution for Use of ICI

ICIs are a versatile class of immunomodulatory agents and have demonstrated clinical benefit in the treatment of several cancers, including metastatic NSCLC. In general, monoclonal Abs, including immunomodulatory Abs, have proven to be well tolerated [35]. The pharmacokinetic (PK) of the approved ICI is similar to that of endogenous immunoglobulin G (IgG). The typical volume of distribution of monoclonal Abs is comparable to the plasma volume (2 to 4 L) [36]. Drug-receptor binding affinity and association–dissociation kinetics have an important role in distribution [37]. Elimination occurs by both specific (target-mediated) and nonspecific (Fc-mediated routes, accounting for the nonlinear and linear elimination PK [36]. Following target saturation, the linear-nonspecific route of elimination is predominant; so, the half-life of ICIs varies from 3 to 4 weeks approximately [36,38].

The first ICI to have been marketed in the US in 2011 was in melanoma; it is ipilimumab. Its administration is intravenous, with an average half-life of 15.4 days [30] (Table 1). The following factors do not represent contraindications to the use because they had no clinically important effect on the clearance of ipilimumab: age, sex, performance status (PS), renal impairment (glomerular filtration rate ≥15 mL/min/1.73 m^2^), or mild hepatic impairment (total bilirubin >1 to 1.5 times the upper limit of normal or aspartate transaminase levels > upper limit of normal) (data available on Bristol Myers Squibb, New York, NY, USA, YERVOY^®^ (ipilimumab)).

Nivolumab is given intravenously, with a mean half-life of 25 days [30] (Table 1). No dose adjustment is recommended in patients with renal failure and mild or moderate hepatic impairment because no side effects are expected under these conditions (data available on Bristol Myers Squibb, OPDIVO™ (nivolumab)).

Pembrolizumab is administered intravenously, with a mean half-life of 22 days [30] (Table 1). No dose adjustment is recommended in patients with renal failure and mild or moderate hepatic impairment (data available on Merck Sharp & Dohme Corp., Kenilworth, NJ, USA, KEYTRUDA^®^ (pembrolizumab)).

Cemiplimab is given intravenously, with a half-life of 20.3 days (Table 1). Mild or moderate renal failure (estimated glomerular filtration rate (eGFR) ≥ 30 mL/min/1.73 m^2^) and mild to moderate liver failure (bilirubin < 3× upper limit of normal and any aspartate transaminase levels) had no clinically significant effect on the systemic exposure of cemiplimab (data available on accessdata.fda.gov with reference ID: 4750303).

Atezolizumab is given intravenously, with a half-life of 27 days [30] (Table 1). Mild or moderate renal failure (estimated glomerular filtration rate (eGFR) ≥ 30 mL/min/1.73 m^2^) and mild to moderate liver failure (bilirubin < 3× upper limit of normal and any aspartate transaminase levels) had no clinically significant effect on the systemic exposure of atezolizumab (data available on Genentech Inc., South San Francisco, CA, USA, TECENTRIQ^®^ (atezolizumab)). 

Durvalumab is administered intravenously, with a half-life of 18 days [30] (Table 1). Mild (creatinine clearance 60 to 89 mL/min) or moderate renal impairment (creatinine clearance 30 to 59 mL/min), and mild hepatic impairment (bilirubin ≤ upper limit of normal and aspartate transaminase levels > upper limit of normal or bilirubin > 1 to 1.5 x upper limit of normal and any aspartate transaminase levels) had no clinically significant effect on the pharmacokinetics of durvalumab (data available on AstraZeneca Pharmaceuticals L.P., Gaithersburg, MD, USA, IMFINZI^®^ (durvalumab)).

ICIs commonly display modest interpatient variability in PK. Some covariates could explain part of the observed variability. They varied between the approved ICIs, but sex, Eastern Cooperative Oncology Group (ECOG) PS, body weight, tumor type, tumor burden, baseline lactate dehydrogenase (LDH), estimated glomerular filtration rate, and albumin can be considered in the differences [39,40,41].

### 4.2. PK and Precaution for Use of Anti-Angiogenic Abs

Bevacizumab is given intravenously, with a half-life of 19.9 days (Table 1). No dose adjustment is recommended in patients with renal failure and mild or moderate hepatic impairment [42].

Ramucirumab is administered intravenously, with a half-life of 14 days and with no dose adjustment in case of renal failure or mild/moderate hepatic impairment [43] (Table 1).

The use of anti-angiogenic molecules is indicated in selected patients only, because of its toxicity. Generally, in subjects with NSCLC, they are safe and well tolerated but some clinical or biological adverse events can interfere with the use of these molecules. Their most common adverse events are hypertension, proteinuria, and epistaxis. Other rarer side effects include neutropenia complications, thromboembolic events, and pulmonary hemorrhage. In order to reduce the incidence of severe hemorrhage, the first randomized clinical trials of bevacizumab in NSCLC excluded subjects with: (1) squamous histology; (2) significant hemoptysis (≥1 teaspoon); (3) tumors invading or abutting major blood vessels or with central tumor localization or with tumor cavitation, based on a radiological assessment; (4) hemorrhagic disorders or in treatment with anticoagulant therapy; (5) brain metastases; (6) ECOG > 1; and (7) age ≥ 75 years [44,45]. However, with the accumulation of real-life data for several years, it seems that the effectiveness and the risk of use of bevacizumab are not affected by the presence of brain metastases, the general condition or age of the patient, or the presence of anticoagulant treatment. To date, only squamous cell histology and a history of hemoptysis deemed clinically significant are absolute contraindications to its use. [46].

## 5. Treatment Paradigm for NSCLC in 2021

The treatment for locally advanced or metastatic NSCLC has changed dramatically in the past 15 years. Since 2007, anti-angiogenic Abs have been used in advanced/metastatic recurrent non-squamous NSCLC [47]. In March 2015, the EMA and FDA approved the first anti-PD-1 antibody as a second line option for treatment of patients with advanced squamous NSCLC [4]. Additionally, a series of inhibitors related to CTLA-4 and PD-1/PD-L1 immune checkpoints have led to the use of immunotherapy for most NSCLC patients, and significantly revised the chemotherapy treatment model. The use of therapeutic Abs (anti-angiogenic or ICI) has become the cornerstone of first line treatment, either as a single modality or with chemotherapy. These advances in lung cancer management, along with targeted agents like tyrosine kinase inhibitors for lung cancer with oncogenic drivers, or lifestyle alterations such as decreasing smoking among men, resulted in a decrease in population-level mortality from NSCLC in the US [48] similar to the EU. We will review the phase II/III clinical trials that led to the marketing authorizations of anti-angiogenic and ICI Abs currently used in NSCLC is the US and in EU and in the US only (Table 2). We will present the indications according to the expression levels of PD-L1, using the same strata as in the clinical trials (≥50%, 1–49% and <1%).

### 5.1. Front Line Treatment in the US and in the European Union

#### 5.1.1. PD-L1 ≥ 50%

Patients with metastatic NSCLC and with PD-L1 > 50% have multiple immunotherapy treatment options. In both the US and EU, monotherapy with pembrolizumab is an approach that may be used, and ICI with chemotherapy could also be given as front line therapy to these patients. In 2021, monotherapy with atezolizumab has also become an option in this setting in the EU.

Pembrolizumab was first used as monotherapy for second line treatment and beyond (see Section below). The KEYNOTE-024 phase III randomized trial included 305 treatment-naïve NSCLC patients with PD-L1 expression in more than 50% of tumor cells and no EGFR/ALK mutations; they were randomized to receive pembrolizumab or platinum-based chemotherapy [9]. Patients treated with pembrolizumab showed better progression-free survival (PFS), overall survival (OS), and overall response rate (ORR) than those treated with chemotherapy [9,49]. Based on these results, the FDA and EMA approved pembrolizumab as a single-agent first line ICI in patients with advanced NSCLC harboring high PDL1 expression (PD-L1 ≥ 50%). The 3-year survival results of KEYNOTE-024 showed that pembrolizumab monotherapy significantly improved the median OS length (26.3 versus 14.2 months) and the 3-year OS rate (43.7% versus 24.9%) compared to chemotherapy. Moreover, the KEYNOTE-042 [50] phase III trial, with the same patient groups, divided each treatment arm into three subgroups based on the level of PD-L1 expression (PD-L1 > 50%, >20%, >1%). All groups showed significantly better OS with pembrolizumab rather than chemotherapy but the OS of patients who received pembrolizumab in the high PD-L1 expression arm (>50%) showed maximum benefit. For this reason, pembrolizumab as monotherapy was restricted to patients with PD-L1 expression ≥ 50%, and not licensed for the group with PD-L1 expression > 1%. 

In 2020, the FDA granted approval to atezolizumab monotherapy as treatment for patients with metastatic NSCLC whose tumors have PD-L1 expression ≥ 50%, without *EGFR/ALK* mutations. In fact, the Impower110 study compared atezolizumab with chemotherapy among patients with high PD-L1 expression [51]. The median OS was 20.1 months with atezolizumab versus 13.1 months with chemotherapy, at a median follow-up of 15.7 months. The EMA approved atezolizumab for the same indication in 2021.

Then it was considered to add an anti-PD-1/PD-L1 ICI to standard chemotherapy in treatment-naïve NSCLC patients, regardless of PD-L1 expression. KEYNOTE-189. KEYNOTE-189 [52] was a phase III trial that assessed first line platinum-based chemotherapy with or without pembrolizumab in *EGFR/ALK*-wild type, non-squamous NSCLC patients. In an updated analysis published in 2020, the median OS was 22 months in the pembrolizumab chemotherapy arm versus 10.7 months in the placebo chemotherapy arm, and the OS advantage was achieved in all PD-L1 subgroups [53]. On the basis of these results, pembrolizumab in combination with pemetrexed and carboplatin as front line treatment in metastatic non-squamous NSCLC became a new standard, regardless of PD-L1 expression. Similar treatment was then evaluated in squamous NSCLC: the KEYNOTE-407 trial randomized patients to receive four cycles of carboplatin and paclitaxel or nab-paclitaxel with or without pembrolizumab [54]. Patients with the pembrolizumab–chemotherapy combined treatment showed a significantly improved OS compared with those with a chemotherapy treatment alone (15.9 versus 11.3 months). The combination with pembrolizumab and platinum-based chemotherapy is therefore used in the front line setting regardless of the level of PD-L1 expression or the tumor histology. 

Another possible treatment option in the US and in the EU for patients with a PD-L1 greater than 50% is a combination of chemotherapy, atezolizumab, and bevacizumab for non-squamous NSCLC. The Impower150 trial randomized patients to three groups: atezolizumab–bevacizumab–carboplatin–paclitaxel (ABCP), atezolizumab–carboplatin–paclitaxel (ACP) and bevacizumab–carboplatin–paclitaxel (BCP). Both the median PFS and OS were improved in the atezolizumab–chemotherapy–anti-angiogenic arm (PFS: 8.3 versus 6.8 months; OS: 19.2 versus 14.7 months) compared with the patients treated with BCP [55]. Therefore, this combination is authorized in the front line setting in the US and in the EU for advanced NSCLC regardless of PD-L1 expression, in the absence of contraindication to bevacizumab. Bevacizumab is the only anti-angiogenic agent approved for the first line treatment of NSCLC in selected patients [56]. 

Recently, another combination of two ICIs and chemotherapy has been approved. The CheckMate-9LA study randomized patients to receive nivolumab plus ipilimumab combined with histology-based, platinum doublet chemotherapy (for two cycles), or chemotherapy alone (for four cycles) [57]. The OS, as well as secondary endpoints of PFS and ORR, were superior for the combination nivolumab plus ipilimumab, given concomitantly with two cycles of chemotherapy, versus chemotherapy alone. This immune–chemotherapy combination is approved regardless of the PD-L1 expression and the tumor histology (Table 2).

#### 5.1.2. PD-L1 1–49%

For patients with PD-L1 between 1% and 49%, treatment options shared by the US and the EU are pembrolizumab and chemotherapy in association regardless of the histology type [52,54], atezolizumab–chemotherapy–bevacizumab only for non-squamous NSCLC [55] and a combination of nivolumab, ipilimumab, and two cycles of chemotherapy for all PD-L1 expressions [57] (Table 2).

#### 5.1.3. PD-L1 < 1%

For patients with PD-L1 < 1%, treatment options shared by the US and the EU are pembrolizumab and chemotherapy in association regardless of the histology type [52,54], or atezolizumab–chemotherapy–bevacizumab only for non-squamous NSCLC [55] (Table 2). In the EU only, the combination of nivolumab, ipilimumab, and two cycles of chemotherapy for all PD-L1 expressions can be used [57].

### 5.2. Front Line Treatment in the US Only 

Most treatment options in the US and in the EU are similar but there are some indications currently specific for the US.

#### 5.2.1. PD-L1 ≥ 50%

Recently, a novel ICI monotherapy has been approved in the US. The EMPOWER-Lung 1 phase III open-label study randomized patients to receive cemiplimab instead of platinum-based chemotherapy. Primary endpoints were assessed in the intention-to-treat population and in a prespecified PD-L1 of at least 50% population [12]. Cemiplimab reduced the risk of death by 43% compared to chemotherapy. This was achieved with a crossover rate to cemiplimab greater than 70% following disease progression on chemotherapy, as well as the largest population of patients with pre-treated and clinically stable brain metastases among advanced NSCLC pivotal trials to date. Therefore, in the US, for these patients with PD-L1 expression ≥ 50%, the choice between pembrolizumab, cemiplimab, and atezolizumab can be made as ICI monotherapy for front line treatment. In Europe, cemiplimab is in the process of being authorized for the same indication.

An ICI combination without chemotherapy is also possible in the US: ipilimumab and nivolumab (see section below) (Table 2).

#### 5.2.2. PD-L1 1–49%

For patients with PD-L1 expression of 1–49%, two additional options are available in the USA compared to the EU. In 2019, the FDA expanded pembrolizumab indication for first line treatment of NSCLC with PD-L1 expression greater than 1%. This approval was based on the KEYNOTE-042 trial [50]: in the PD-L1 ≥ 1% population (overall population), the median OS was 16.7 and 12.1 months for the pembrolizumab and chemotherapy arms, respectively. In the ≥ 50% subgroup, the estimated median OS was 20 months and 12.2 months for those receiving pembrolizumab and chemotherapy, respectively. There were no significant differences in progression-free survival or overall response rate between arms in any population.

The second additional option compared to the EU is the ICI combination nivolumab and ipilimumab, based on the CheckMate227 study [6,58]. This trial recruited treatment-naïve patients with advanced NSCLC. They were randomized to receive nivolumab plus ipilimumab, nivolumab with or without chemotherapy, and histology-based chemotherapy arms [6,58]. According to PD-L1 expression, patients were enrolled into PD-L1 > 1% and <1% cohorts, and further randomized 1:1:1 to nivolumab plus ipilimumab, platinum-based chemotherapy, or nivolumab monotherapy (PD-L1 > 1% group), or nivolumab plus platinum-based chemotherapy (PD-L1 < 1% group). The study protocol was later modified to include a co-primary endpoint of PFS in patients with high tumor-mutational burden (TMB) (defined by ≥10 mutations per megabase). Nivolumab plus ipilimumab combined treatment showed a significantly prolonged PFS than for the chemotherapy arm among patients with high TMB (7.2 versus 5.5 months), but median OS was not significantly different between these groups. The final analysis for Checkmate-227 was based on the PD-L1 ≥ 1% cohort and demonstrated a significantly higher median OS in patients who received ipilimumab plus nivolumab than in patients treated with chemotherapy alone (median OS: 17.1 months vs. 14.9 months) [58]. Median PFS was also improved with nivolumab plus ipilimumab over chemotherapy for all patients regardless of PD-L1 expression, but was numerically higher for patients in the PD-L1 expression < 1% cohort. The final analysis did not demonstrate improved OS based on TMB. In CheckMate568, the association of efficacy with PD-L1 expression and TMB was assessed in patients who received first line nivolumab plus ipilimumab [59]. Higher response rates and improved PFS were observed in patients with TMB of ≥ 10 mutations per megabase versus TMB < 10 mutations per megabase, irrespective of PD-L1 expression. Currently, nivolumab and ipilimumab combination therapy is approved in the US based on PD-L1 expression alone, for patients with PD-L1 expression > 1% (Table 2).

#### 5.2.3. PD-L1 < 1%

There is no difference in therapeutic strategy for patients with PD-L1 expression less than 1% (Table 2).

#### 5.2.4. Patients Who Are Not Eligible to Receive ICI 

If first-line ICI cannot be used due to contraindications or active autoimmune disease, standard platinum-based doublet therapy is often given. The addition of bevacizumab to chemotherapy can also be discussed in eligible patients. Bevacizumab was evaluated in 2006 in a phase III clinical with stage IIIB-IV non-squamous NSCLC patients [19]. Patients were treated with carboplatin–paclitaxel for six cycles with or without bevacizumab at 15 mg/kg. Bevacizumab could then be administered as maintenance therapy with continuation until signs of disease progression or unacceptable toxicity appeared. OS was significantly higher in bevacizumab group (12.3 versus 10.3 months) and PFS was increased too. The same results were observed in the AVAiL trial with chemotherapy by cisplatin and gemcitabine, with or without bevacizumab, for the two doses tested (7.5 or 15 mg/kg) [20]. After that, the phase III POINTBREAK trial randomized patients in two arms: pemetrexed–carboplatin–bevacizumab followed by pemetrexed plus bevacizumab in maintenance or paclitaxel–carboplatin–bevacizumab followed by maintenance therapy with bevacizumab alone [60]. The primary endpoint of OS did not reach statistical significance, although in the group treated with pemetrexed and bevacizumab in maintenance therapy, an increased PFS was reported. The AVAPERL study was another phase III trial that randomized patients after four cycles of chemotherapy with cisplatin–pemetrexed–bevacizumab, to receive maintenance treatment with bevacizumab alone or with pemetrexed and bevacizumab [61]. The PFS was improved in the double maintenance group (10.2 versus 6.6 months) and the result for OS was the same, but it was not statistically significant.

#### 5.2.5. VEGF/VEGFR Antibody Therapy in Patients with EGFR Mutations 

TKI are standard treatment options for NSCLC with EGFR mutations. Some preclinical studies had shown that the *EGFR* signaling pathway can upregulate VEGF expression [62] and that VEGF/VEGFR can play a role in EGFR-TKI resistance [63]. This led to the investigation of combination treatments with EGFR-TKIs and anti-angiogenic Abs. 

In the EU, bevacizumab in combination with erlotinib has been indicated for first line treatment of adult patients with unresectable advanced, metastatic, or recurrent NSCLC with *EGFR* mutations since 2016. It is recommended that the treatment with bevacizumab in addition to erlotinib is continued until disease progression. The pivotal JO25567 study was a randomized phase II study conducted to assess the safety and efficacy of first line bevacizumab in combination with erlotinib compared to erlotinib alone [64] in Japanese patients with advanced NSCLC with *EGFR* mutations. Seventy-seven patients were randomly assigned to receive erlotinib and bevacizumab and 77 to receive erlotinib alone. Median PFS was 16.0 months with erlotinib plus bevacizumab and 9.7 months with erlotinib alone. OS was not improved in patients receiving the combination. The safety analysis population comprised 75 patients in the erlotinib plus bevacizumab group and 77 in the erlotinib group, who received at least one dose of the study drug. There was no difference in incidence of serious adverse events between the two groups. Nevertheless, more grade > 3 adverse events were observed with the erlotinib–bevacizumab combination (90.7%) than with erlotinib alone (53.2%). These side effects were primarily grade 3 hypertension, but it was manageable with antihypertensive drugs in most cases. Other side effects were reported such as the presence of proteinuria or bleeding, but which remained manageable and which did not lead to early discontinuation of treatment [65]. 

More recently, in 2020, the FDA and EMA approved ramucirumab in combination with erlotinib for first line treatment of metastatic NSCLC with *EGFR* mutations. Efficacy was evaluated in the RELAY trial, a multinational, randomized, double-blind, placebo-controlled, worldwide, multicenter study in patients with previously untreated metastatic NSCLC whose tumors had *EGFR* exon 19 deletion or exon 21 (L858R) substitution mutations [23]. A total of 449 patients were randomized (1:1) to receive either ramucirumab 10 mg/kg or placebo every 2 weeks, in combination with erlotinib once daily, until disease progression or unacceptable toxicity. Median PFS was 19.4 months in the ramucirumab plus erlotinib arm compared with 12.4 months in the placebo plus erlotinib arm. ORR was 76% in the ramucirumab plus erlotinib arm and 75% in the placebo plus erlotinib arm, with median duration of response of 18.0 months and 11.1 months, respectively. The follow-up was not sufficient to determine whether the combination led to an OS benefit. The most common adverse reactions observed in patients treated with ramucirumab with erlotinib were hypertension (24%), dermatitis acneiform (15%), alanine aminotransferase increase (8%), and diarrhea (7%).

### 5.3. Second Line Treatment and Beyond in the European Union and the US 

Second line treatment for metastatic lung cancer is determined by the type of agents received in the first line setting. There are some challenges in second line treatment: patients are pre-treated, often with reduced PS, thus tolerability becomes more important; responses rates are lower; thus, tumor control becomes important; and patients can be more symptomatic, thus symptom control and symptom improvement become important. 

Most patients who can receive ICI therapy are treated with first line immunotherapy with or without chemotherapy, and ICI re-challenge is not a standard second line treatment. Ramucirumab is an anti-angiogenic Ab that can be used in the second line setting of NSCLC. The REVEL study was a phase III, placebo-controlled trial which included patients with metastatic NSCLC who had progressed during or after platinum-based chemotherapy, with or without bevacizumab [22]. Patients were randomly allocated to receive docetaxel and either ramucirumab or placebo. Median OS was 9.1 months versus 10.5 months in the placebo and ramucirumab group, respectively. The addition of ramucirumab to docetaxel can be considered in younger patients with good PS as a second line treatment.

Using a therapeutic strategy containing bevacizumab or ramucirumab is feasible in selected patients because of the efficacy and safety related to the administration of these Abs [66]. Ramucirumab was safe across all NSCLC histologies in the second line setting. The anti-angiogenic Ab bevacizumab is only used in non-squamous NSCLC because of more frequent and severe pulmonary hemorrhage in patients with squamous NSCLC, as shown in a phase II trial in 2004 [67]. In fact, squamous cell tumors are more likely centrally located and cavitated compared to adenocarcinoma; but it is not clear whether the histology, as is, is an independent risk factor or a marker of increased risk. Another problem was the presence of brain metastasis, at risk of bleeding: a factor long considered as an exclusion criterion for treatment with bevacizumab. A retrospective analysis of 17 studies on the use of bevacizumab in patients with untreated metastases for lung, breast, kidney, or colorectal cancer showed that the use of bevacizumab did not increase risk of bleeding [68]. The BRAIN phase II trial enrolled patients with untreated brain metastases and the rate of central nervous system hemorrhage was comparable to that of a previous bevacizumab study, in which the presence of untreated brain metastases was an exclusion criteria [69]. Without detailing all the studies supporting these results, we can say that many of the factors previously considered as exclusion criteria, such as anticoagulant treatment, central tumor location, the presence of cavitation or brain metastases, and an advanced age, are not currently valid [45,46,70,71]. In fact, the only exclusion criteria that are absolute contraindications to the use of bevacizumab are squamous histology and the presence of hemoptysis [45]. 

If chemotherapy alone has been prescribed in the first line, monotherapy with ICI may be considered. As a second line therapy for stage IIIB or IV squamous NSCLC with disease recurrence after one prior platinum-containing regimen, in a phase III study, CheckMate 017, nivolumab was compared to docetaxel [4]. Nivolumab was shown to be more efficacious than chemotherapy: OS was improved in the ICI group (9.2 versus 6 months), as well as PFS (3.5 versus 2.8 months). Subsequently, the CheckMate 057 phase III study randomized patients with a metastatic non-squamous NSCLC in two groups: nivolumab versus docetaxel [7]. Nivolumab demonstrated superior OS (12.2 versus 94 months) despite a lower median PFS (2.3 versus 4.2 months). Following these results, nivolumab was approved by the FDA and EMA as second line therapy for metastatic NSCLC with progression on or after standard chemotherapy. PD-L1 testing was not required for nivolumab administration. This molecule was therefore the first ICI used in thoracic oncology.

Unlike nivolumab, which received non-restricted FDA and EMA approval, pembrolizumab received accelerated approval with companion diagnostic PD-L1 assay of 1% or more for advanced NSCLC that progressed during or after front line chemotherapy. The KEYNOTE-010 study recruited patients with NSCLC in a second line setting in order to evaluate two dosing regimens for pembrolizumab (2 and 10 mg/kg) compared to docetaxel [8]. OS was improved in the pembrolizumab group, at both doses tested, compared to chemotherapy: OS was 10.4 and 12.7 months for the 2 and 10 mg/kg pembrolizumab doses respectively, compared with 8.5 months for the docetaxel group. In contrast, the differences in PFS were not statistically significant between the different dose pembrolizumab groups and docetaxel group. Thus, in 2016, the EMA and FDA approved pembrolizumab for treatment of patients with metastatic NSCLC whose tumors express PD-L1 greater than ≥1%) with disease progression on or after platinum-containing chemotherapy. Previous analyses indicated that the observed pembrolizumab exposures in patients with squamous cell carcinoma of the head and neck treated with pembrolizumab at fixed dose (200 mg every three weeks) were similar to the pembrolizumab exposure data observed in patients with other solid tumors (mainly melanoma and NSCLC) receiving a weight-dependent dose of pembrolizumab (2 mg/kg every three weeks), supporting a fixed 200 mg every three weeks dose for the head and neck cancer indication [72]. Based on these data, the FDA and EMA recommended also a fixed dose of pembrolizumab (200 mg) for the treatment of patients with metastatic NSCLC, irrespective of prior line of therapy.

The third monotherapy ICI available is atezolizumab, approved in 2016 by the FDA and EMA for the treatment of patients with NSCLC whose cancer had progressed during or after standard chemotherapy. This approval was based on two trials, a phase II POPLAR [15] and phase III OAK study [14]. The randomized OAK trial compared atezolizumab to docetaxel and found a median OS of 13.8 months versus 9.6 months in the chemotherapy group.

### 5.4. Locally Advanced Inoperable NSCLC (IIIA/B/C Stages) 

In patients with stage III NSCLC that is not resectable, ICI has been considered. According to the PACIFIC study [13], durvalumab is given for 12 months if no progression after concomitant radio–chemotherapy is seen, but only if PD-L1 > 1% in some countries. In the US and France, durvalumab is allowed in patients with a PD-L1 < 1%.

### 5.5. Treatment Decision Algorithm in the European Union and in the US

For the use of ICI, contraindications to those molecules should always be kept in mind (uncontrolled autoimmune pathologies, uncontrolled HIV–HBV–HCV infections, for example) but this is to be discussed on a case-by-case basis, during multidisciplinary meetings [70].

**Figure 2:**
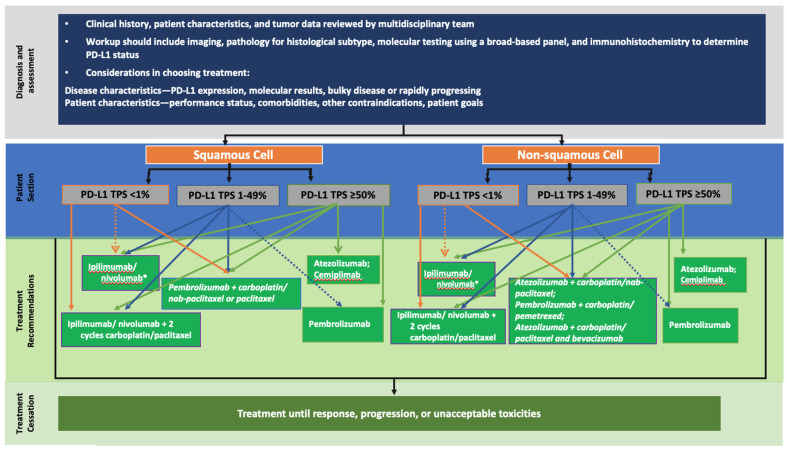
Potential treatment paradigm in the US for NSCLC without actionable mutations in front line. Adapted from [71,73]. * Not FDA approved for PD-L1 < 1%.

**Figure 3:**
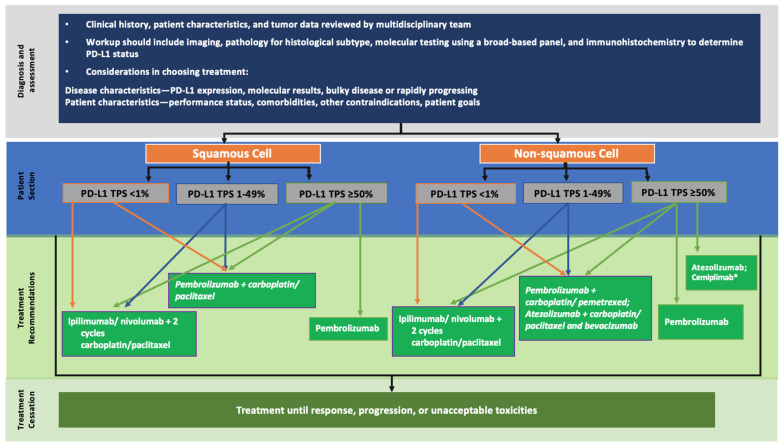
Potential treatment paradigm in the European Union for NSCLC without actionable mutations in front line. Adapted from [71,73]. * European authorization expected shortly.

In the front line setting, the first selection is then made based on the expression of PD-L1 (Figure 2 and Figure 3). In chemotherapy–immunotherapy trials, patients had a PS of 0 or 1 and a median age of 65 years [52,54]. Therefore, the pembrolizumab + chemotherapy regimen is mostly reserved for patients who are not too old and in good general condition. However, we observe the same data in the first line immunotherapy alone studies (PS 0–1 and median age 65 [50]); in practice, immunotherapy as monotherapy seems to have better tolerance and is therefore to be favored in older patients and those in less good general condition. In any case, there is no restriction of approvals and marketing authorizations in the EU and in the US based on age or PS. Finally, in case of non-squamous NSCLC, the use of the quadruple combination ABCP must include verification that contraindications to anti-angiogenic agents do not exist [46]. Regarding the nivolumab–ipilimumab–chemotherapy combination, the study group included 70% non-squamous NSCLC, 40% PD-L1 negative and 20% hepatic metastases [57]; factors should be considered to reserve this combination for certain types of patients (Figure 2 and Figure 3).

In the second line, the treatment is directly dictated in part by the treatment received in the front line. If chemotherapy as monotherapy has been used, regardless of PD-L1 expression, atezolizumab or nivolumab as monotherapy may be considered, in the absence of contraindications to ICI (Figure 2 and Figure 3). Again, the trials included 0–1 PS patients with a median age of 65 years. The OAK study using atezolizumab [14] included nearly 50% of patients over 65 years of age and 10% of them had stable brain metastases. The Checkmate studies with nivolumab [4,7] included 41% of patients over 65 in the nivolumab arm but 93% had no brain metastases. The center-specific experience can then also be considered to choose between these two ICIs. The expression of PD-L1 is not restrictive for the prescription of these two molecules, unlike pembrolizumab, where the tumor must express more than 1% of PD-L1 (Figure 2 and Figure 3). The keynote studies [9] included 15% of patients with stable brain metastases. The clinician is therefore free to choose ICI as monotherapy in the second line if the patient did not receive it in the front line setting and does not have contraindications.

If an ICI has been used as a first line monotherapy, it is possible to treat the patient with a chemotherapy and anti-angiogenic combination. This in the absence of contraindications to bevacizumab [46] and for non-squamous NSCLC only [66]: the regimen is then platinum-based chemotherapy, pemetrexed or paclitaxel, and bevacizumab. If platinum-based chemotherapy has been used in first line with an ICI, the second line chemotherapy association could be paclitaxel–bevacizumab. Finally, a last possible chemotherapy anti-angiogenic Ab combination is docetaxel with ramucirumab [22] for both non-squamous and squamous NSCLC (Table 1).

## 6. Conclusions

The field of antibodies as anti-cancer treatment has led to anti-angiogenic and immunotherapeutic Ab use for most patients with advanced NSCLC. The agents have developed rapidly since the first US FDA and EMA approvals in 2007 and 2015 of anti-angiogenic and ICI Abs respectively. Though initially available in the second line setting, ICI expanded into the first line setting and increased the therapeutic options and combinations used for metastatic NSCLC patients without driver mutations. The treatment of these patients on both sides of the Atlantic is generally similar, with some broader indications in the US. In fact, a study published in 2017 confirmed that the FDA approves more drugs, at a faster rate, than the EMA [74]. This is probably related to the different evaluation criteria used by the two regulatory authorities, but also to the differences in the health insurance system and reimbursement constraints. Now that most patients can benefit from treatment in the first or second line setting with ICI, the key for the years to come is the determination of predictive biomarkers of response to identify the best individual strategy for each patient.

## Figures and Tables

**Table 1:** Characteristics of marketed antibodies in NSCLC. According to [30]. * European authorization expected shortly.

Antibody	Target	Administration	Indication in US	Indication in European Union	Ig Class	Half-Life (Days)	Dose in the US	Dose in European Union	Interval between 2 Injections
Ipilimumab	CTLA-4	Intravenous	front line with nivolumab alone (PD-L1 ≥ 1%) or + 2 cycles platinum-based chemotherapy	front line with nivolumab + 2 cycles platinum-based chemotherapy	IgG1	15.4	1 mg/kg (with nivolumab)	1 mg/kg (with nivolumab)	6 weeks (with nivolumab +/− chemotherapy)
Nivolumab	PD-1	Intravenous	front line with ipilimumab alone (PD-L1 ≥ 1%) or + 2 cycles platinum-based chemotherapy Second line	front line with ipilimumab + 2 cycles platinum-based chemotherapy Second line	IgG4	25	3 mg/kg (with ipilimumab) 360 mg (with chemotherapy + ipilimumab) 240 or 480 mg (monotherapy)	3 mg/kg (with ipilimumab) 360 mg (with chemotherapy + ipilimumab) 240 mg (monotherapy)	2 weeks 3 weeks (with chemotherapy + ipilimumab) 2 weeks (240 mg) 4 weeks (480 mg)
Pembrolizumab	PD-1	Intravenous	front line alone (PD-L1 ≥ 50% or PD-L1 ≥ 1%) or with platinum and pemetrexed (non-squamous NSCLC); with carboplatin and paclitaxel or nab-paclitaxel (squamous NSCLC) second line (PD-L1 ≥ 1%)	front line alone (PD-L1 ≥ 50%) or with platinum and pemetrexed (non-squamous NSCLC); with carboplatin and paclitaxel or nab-paclitaxel (squamous NSCLC) Second line (PD-L1 ≥ 1%)	IgG4	22	200 or 400 mg (monotherapy or with chemotherapy)	200 mg (monotherapy or with chemotherapy) 400 mg (in maintenance and if well tolerated)	3 weeks (200 mg) 6 weeks (400 mg)
Cemiplimab	PD-1	Intravenous	front line alone (PD-L1 ≥ 50%)	front line alone (PD-L1 ≥ 50%) *	IgG4	20	350 mg		3 weeks
Atezolizumab	PD-L1	Intravenous	front line alone (PD-L1 ≥ 50%) or with carboplatin, paclitaxel and bevacizumab (non-squamous NSCLC); or with carboplatin and nab-paclitaxel (non-squamous NSCLC) second line	front line alone (PD-L1 ≥ 50%) or with carboplatin, paclitaxel and bevacizumab (non-squamous NSCLC); Second line	IgG1	27	840 mg or 1200 mg (with chemotherapy) or 1680 mg	1200 mg	2 weeks (840 mg) 3 weeks (with chemotherapy or 1200 mg) 4 weeks (1680 mg)
Durvalumab	PD-L1	Intravenous	consolidation following concurrent chemoradiation for unresectable stage III NSCLC	consolidation following concurrent chemoradiation for unresectable stage III NSCLC	IgG1	18	10 mg/kg or 1500 mg	10 mg/kg	2 weeks (10 mg/kg) or 4 weeks (1500 mg)
Bevacizumab	VEGF	Intravenous	front line with carboplatin and paclitaxel (non-squamous NSCLC) or with atezolizumab, carboplatin and paclitaxel (non-squamous NSCLC)	front line with carboplatin and paclitaxel (non-squamous NSCLC) or with atezolizumab, carboplatin and paclitaxel (non-squamous NSCLC) or with erlotinib (if *EGFR* mutation)	IgG1	19.9	15 mg/kg	7,5 or 15 mg/kg (with cisplatin chemotherapy) 15 mg/kg (with carboplatin chemotherapy)	3 weeks
Ramucirumab	VEGFR	Intravenous	front line with erlotinib (if *EGFR* mutation) second line with docetaxel	front line with erlotinib (if *EGFR* mutation) second line with docetaxel	IgG1	14	10 mg/kg	10 mg/kg	2 weeks (with erlotinib); 3 weeks (with docetaxel)

**Table 2:** Algorithm for front line immune checkpoint inhibitors’ treatment of patients with NSCLC based on PD-L1 expression.

Sub-Group	Treatment in the US	Comments	Treatment in European Union	Comments
PD-L1 ≥ 50%	pembrolizumab single agent		pembrolizumab single agent	
	atezolizumab single agent		atezolizumab single agent	
	cemiplimab single agent		cemiplimab single agent *	
	pembrolizumab ^2^ + chemotherapy^1^		pembrolizumab ^2^ + chemotherapy ^1^	
	atezolizumab ^4^ + chemotherapy ^3^ +/− bevacizumab ^5^	Non-squamous NSCLC only	atezolizumab ^4^ + chemotherapy ^3^ + bevacizumab ^5^	Non-squamous NSCLC only
	nivolumab + ipilimumab			
	nivolumab + ipilimumab + chemotherapy ^7^		nivolumab + ipilimumab + chemotherapy ^7^	
PD-L1 1–49%	pembrolizumab single agent		-	
	pembrolizumab ^2^ + chemotherapy ^1^		pembrolizumab ^2^ + Chemotherapy ^1^	
	atezolizumab ^4^ + chemotherapy ^3^ +/− bevacizumab^5^	Non-squamous NSCLC only	atezolizumab ^4^ + chemotherapy ^3^ + bevacizumab ^5^	Non-squamous NSCLC only
	nivolumab + ipilimumab		-	
	nivolumab + ipilimumab + chemotherapy ^6^		nivolumab + ipilimumab + chemotherapy ^6^	
PD-L1 < 1%	pembrolizumab ^2^ + chemotherapy ^1^		pembrolizumab ^2^ + Chemotherapy ^1^	
	atezolizumab ^4^ + chemotherapy ^3^ +/− bevacizumab ^5^		atezolizumab ^4^ + chemotherapy ^3^ + bevacizumab ^5^	Non-squamous NSCLC only
	nivolumab + ipilimumab + chemotherapy ^6^		nivolumab + ipilimumab + chemotherapy ^6^	Awaiting a recommendation in first semester 2021

^1^ Chemotherapy is cisplatin or carboplatin + pemetrexed for non-squamous NSCLC. Chemotherapy is carboplatin + paclitaxel for squamous NSCLC. ^2^ Continuation maintenance until progression, unacceptable toxicity, with pemetrexed in non-squamous NSCLC. ^3^ Chemotherapy is carboplatin + paclitaxel. ^4^ Continuation maintenance until progression, unacceptable toxicity. ^5^ Double continuation maintenance with bevacizumab–atezolizumab until progression or unacceptable toxicity. ^6,7^ Chemotherapy is cisplatin or carboplatin + pemetrexed for non-squamous NSCLC. Chemotherapy is carboplatin + paclitaxel for squamous NSCLC. Two cycles of chemotherapy.* European authorization expected shortly.

## Data Availability

Data sharing not applicable.
